# The effect of regular aerobic exercise on sleep quality and fatigue among female student dormitory residents

**DOI:** 10.1186/s13102-020-00190-z

**Published:** 2020-08-05

**Authors:** Maryam Ezati, Maryam Keshavarz, Zahra Amirkhanzadeh Barandouzi, Ali Montazeri

**Affiliations:** 1 Department of Midwifery and Reproductive Health, Faculty of Nursing and Midwifery, Iran University of Medical Sciences, Tehran, Iran; 2 School of Nursing, University of Connecticut, Storrs, CT USA; 3 Population Health Research Group, Health Metrics Research Center, Iranian Institute for Health Sciences Research, ACECR, Tehran, Iran

**Keywords:** Aerobic exercise, Sleep quality, Fatigue, Dormitory students

## Abstract

**Background:**

Emerging research shows a high prevalence of fatigue and sleep problems among university students. The present study evaluates the effects of regular aerobic exercise on sleep quality and fatigue level among female students (ages 18–26) who reside in dormitories.

**Methods:**

This quasi-experimental study involving 67 participants consisted of one experimental group (i.e., assigned aerobic exercise) and one control group (i.e., not assigned aerobic exercise). Participants in the experimental group received three one-hour sessions aerobic exercise weekly ranging from mild to moderate intensity for eight-week. Sleep quality and fatigue level were evaluated using the Pittsburgh Sleep Quality Index (PSQI) and standard Multidimensional Fatigue Inventory (MFI-20), respectively. These variables were assessed at baseline, week four, and week eight of the study.

**Results:**

After four and 8 weeks of the intervention, participants in the aerobic group showed improvement in the score of sleep quality (*p* < 0.001 and *p* < 0.0001, respectively) and its components (except for sleep duration after 4 weeks intervention). Also, aerobic exercise resulted in a significant reduction of the total score of fatigue and its dimensions in weeks four and eight, compared to the control group (*p* < 0.001).

**Conclusions:**

Four-week aerobic exercise with mild intensity had no significant effect on sleep duration. Conversely, intensified aerobic exercise for 8 weeks influenced all components of sleep quality.

**Trial registration:**

The study was registered on 6/2/2015 in the Iranian Registry of Clinical Trials (IRCT) with number IRCT201412282324N15.

## Background

Sleep is a biological process that is necessary for optimal neurologic function, as well as systematic biology, including metabolism, appetite regulation, immunity, hormonal balance, and cardiovascular system [1, 2]. Sleep disorders affect a considerable number of people globally and may be increasing in prevalence [3]. University students with sleep problems may also experience a decline in health and academic performance [4]. In different surveys in the United States, the prevalence of poor sleep quality has reportedly been between 27 and 60% of university students [4, 5]. In one study, the prevalence of sleep disorders in Iranian University students was reported by 40.6% [6]. It appears that residency in dormitories has been cited as an influential factor that affects sleep quality in students [7]. Results of a survey from 23 countries revealed that physical activity was below the recommended levels for the majority of university students. More than 50% of American university students are not sufficiently active [8]. Also, there is a growing concern for the physical inactivity of Iranian university students [9].

The American Academy of Sleep Medicine (AASM) recommends regular physical activity for proper sleep hygiene. AASM suggests exercise can be a non-pharmacological intervention for sleep quality improvement [10]. In a systematic review of 34 studies, 29 studies reported a positive effect of physical activity on sleep quality and its duration among all age groups [11]. Although numerous studies reported the effectiveness of exercise on sleep quality of people in different age ranges across various chronic conditions [12–19], there are contradictory results regarding the impact of exercise on the sleep quality of university students, especially those that reside in dormitories [20–24]. In a quasi-experimental study conducted in Iran, exercise did not have a significant effect on the sleep quality of students [20]. Some studies have shown that gender is an indicator of sleep quality [25], with women reportedly suffering from sleep dysfunction more than men [21, 25]. The intensity of exercise is another factor that was evaluated in limited studies [18, 23].

Daily fatigue is one of the consequences people with sleep dysfunction deal with [26]. While the effect of exercise on fatigue has been evaluated in chronic diseases [27, 28], there are few studies on young adults, as well as in dormitory students. The present study was designed to investigate the effects of an eight-week aerobic exercise intervention on sleep quality and daily fatigue. It was hypothesized that regular aerobic exercise with increasing intensity would improve sleep quality and decrease fatigue level among female students living in dormitories.

## Methods

### Study design and sampling

This study was conducted from October to December 2015. Two out of eight dormitories at the Iran University of Medical Sciences (IUMS) were randomly selected. One of the randomly selected dormitories was the experimental group, whereas the second served as the control group. A non-probability sampling method was used to enroll participants in each of the two selected dormitories. The use of quasi-experimental design minimized the influence of confounding covariates such as students’ interactions regarding the intervention and the process of the study.

### Participants

Forty eligible volunteers were enrolled from each of the two dormitories. Participants were Iranian females between 18 and 26 years of age who met the following criteria: BMI < 29 (kg/m2), non-smoking, no use of acupuncture or other complementary medicines over the last 6 months, and no exposure to stressful events over the past 3 months. They were not enrolled or later dropped out based on the following criteria: a presence of physical or mental illness or surgical history, unwillingness to continue the study, absent for three consecutive or five non-consecutive exercise sessions, involvement in daily physical activity beyond the study protocol, the use of complementary medicines or herbal therapy that influences sleep or fatigue level (i.e., energy-enhancing drugs and/or sleeping medications).

### Tools

The Pittsburgh Sleep Quality Index (PSQI) was used to measure sleep quality [29]. This tool consists of seven areas including: subjective sleep quality, sleep latency, sleep duration, sleep efficiency (the percentage of sleeping time during the time in bed), sleep disturbances (night time waking), use of sleeping medications, and daytime dysfunction (distress and impaired daytime functioning). Scoring of answers was based on a scale of 0 to 3 (total score of 21). Higher scores indicate poorer sleep quality [29]. In the present study, all participants received a score of zero for area six (the use of sleeping medications) since we did not allow students who took sleeping medication to participate. Several studies have evaluated the validity and reliability of the PSQI as a measurement for sleep quality [30–32]. In one Iranian study, the reliability of the Persian version of the PSQI had a Cronbach’s alpha coefficient of 0.77 [33]. In our sample, the PSQI Cronbach’s alpha coefficient was 0.80.

The Multidimensional Fatigue Inventory (MFI-20) was used to assess the fatigue level. This instrument consists of a 20-item survey that covers the following five dimensions: general fatigue (impairment of overall daytime functioning), physical fatigue (body tiredness), mental fatigue (fatigue related to cognition), reduced activity, and reduced motivation. The score of each dimension ranges from 4 to 20. The composite score of fatigue level is the sum of the five dimensions’ scores (i.e., composite score ranges from 20 to 100). Higher scores indicate greater fatigue. The validity and reliability of MFI-20 have been tested previously [34]. The reliability of the Persian version of MFI-20 was evaluated in Multiple Sclerosis [35]. The reliability of the MFI-20 in the present sample resulted in a Cronbach’s alpha coefficient of 0.85.

### Study procedure

Following a discussion of the research objectives and informed consent, the participants in both experimental and control groups individually filled out a demographic questionnaire, the PSQI, and the MFI- 20.

**Fig. 1:**
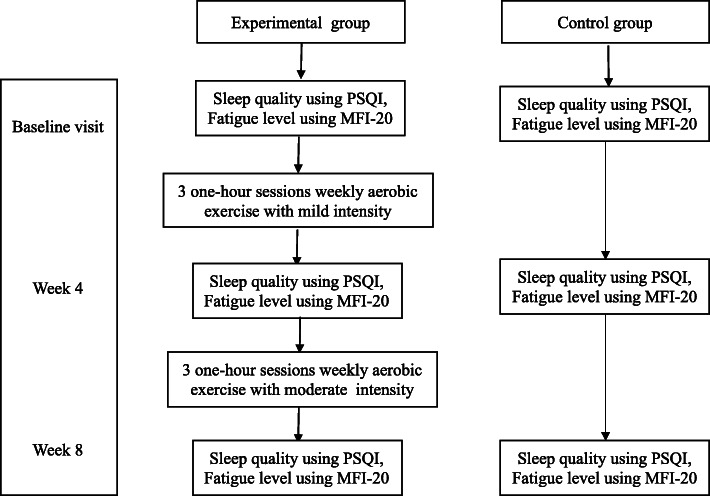
Study procedure flow chart. PSQI, Pittsburgh Sleep Quality Index; MFI-20, Multidimensional Fatigue Inventory-20

At the beginning of each session, a German Buere digital heart rate display was worn around the wrist of the participants. The participants in the experimental group collectively received aerobic exercise plan from an exercise specialist in the dormitories’ gym. The participants exercised with the trainer during three one-hour sessions weekly for eight consecutive weeks. Each session included a 10 min warm-up (marching, top-to-bottom movements), 35 min of basic aerobic exercises (10 min combined exercises, 20 min of mental activity, 5 min mat exercise), and a 15 min cool-down. The schedule was from 17:00 to 18:00 pm. The subjects performed the exercises at 45–50% of maximum heart rate (mild intensity) during the first 4 weeks of intervention and at 65–70% of maximum heart rate (moderate intensity) during the second 4 weeks of the intervention [16]. Intensities were estimated using Karvonen’s formula (i.e., maximum heart rate minus the subject’s mean age [31]. At the end of the fourth and eighth weeks of the intervention, all participants in the study filled out the PSQI and MFI-20 questionnaires. Students in the control group did not receive any intervention and performed their daily routines (Fig. 1).

### Sample size

The sample size was estimated based on the expected difference in study outcomes between the experimental and control groups. To be able to detect a 25% difference in sleep quality between two groups, a study with a sample size of 72 would be required (36 per each group). As such, the study would have a power of 80% at a 5% significance level. The effect size was assumed to be 1. However, this study recruited 40 students for each group to avoid follow-up losses.

### Statistical tests

Results were analyzed using SPSS version 25. Kolmogorov-Smirnow was used to test the normality of the variables. All variables were identified as non-normal (except BMI). The Mann–Whitney test was utilized for variables with non-normal distribution. An independent sample T-test was utilized for BMI. To compare the qualitative data, Chi-square and the exact Fisher test were used. Friedman’s test was utilized to compare each component of sleep quality at different time periods in each group.

## Results

**Fig. 2:**
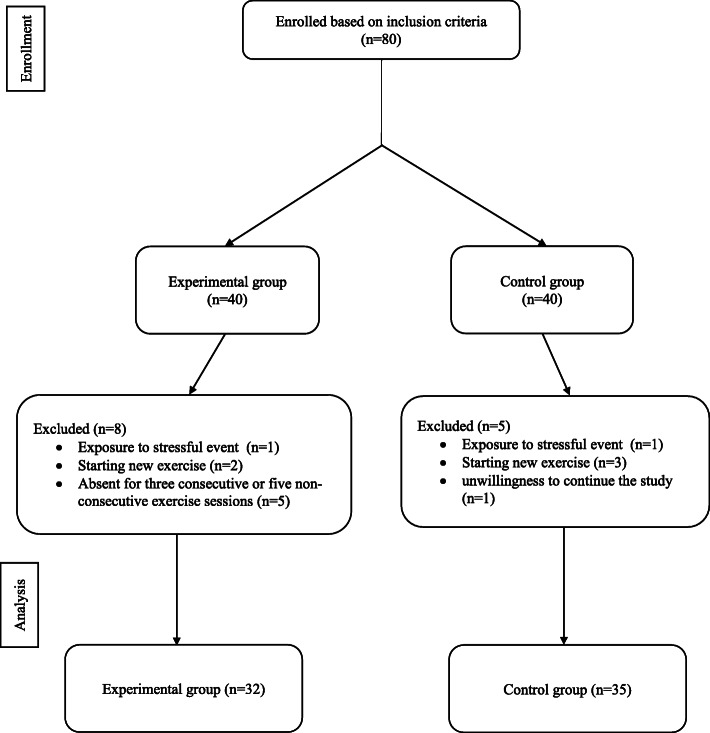
Consort flow diagram

A total of 67 participants, 32 participants in the experimental group and 35 participants in the control group, completed this eight-week study (Fig. 2). There were no significant differences in the demographic characteristics (Table 1), sleep quality (Table 2), and fatigue level (Table 3) between the two groups at baseline. After four and 8 weeks of aerobic exercise, the total scores for sleep quality and its components (except for sleep duration after 4 weeks intervention) were significantly lower than the control group (Table 2). Aerobic exercise resulted in a significant reduction of the total score of fatigue level and its dimensions in weeks four and eight compared to the control group (Table 3). The results of the Friedman test indicated significant improvement in all components of sleep quality and fatigue level in the treatment group after completion of the intervention (Tables 2 and 3). Moreover, we observed a significant increase in subjective sleep quality, daytime dysfunction, and the global PSQI scores in the control group over time (Table 2).

**Table 1:** Characteristics of the subject’s demographics in the two groups

	Experimental group (*n* = 32)	Control group (*n* = 35)	*p-value*
Age (year)	20.53 ± 1.60	20.08 ± 1.31	*0.25
BMI (Kg/m2)	21.82 ± 2.11	22.78 ± 2.34	**0.17
Occupational status (n)^a^ (Employed, unemployed)	(6, 26)	(8, 27)	***0.68
Household economic (n) status (Good, Average)	(30, 2)	(32, 3)	***0.17
Program of study			
Nursing & Midwifery	21.88%	34.3%	***0.31
Management & Statistics	43.75%	37.14%	
Nutrition & Health	25%	17.14%	
Others	9.37%	11.42%	

* Mann Whitney

** Independent sample T-test

*** Fisher exact test

^a^ (n) Number

**Table 2:** Effect of the aerobic exercise intervention on sleep, as measured by the Pittsburgh Sleep Quality Index

Variable	Groups	Baseline			4 weeks of Intervention			8 weeks of Intervention			Friedman
		Mean ± SD^a^	Median (CI)^b^	*p*-value	Mean ± SD	Median (CI)	*p*-value	Mean ± SD	Median (CI)	*p*-value*	*p*-value**
Subjective sleep quality	Experimental	0.93 ± 0.06	0 (1–1)	*0.27*	0.53 ± 0.50	1 (0–2)	*< 0.001*	0.25 ± 0.43	0 (0–0)	< 0.001	*< 0.001*
	Control	0.77 ± 0.64	1 (0–1)		1.14 ± 0.64	1 (1–1)		1.40 ± 0.65	1 (1–2)		*< 0.001*
Sleep duration	Experimental	1.34 ± 0.90	1 (1–2)	*0.16*	0.81 ± 0.64	1 (1–1)	*0.09*	0.40 ± 0.49	1 (0–1)	< 0.001	*< 0.001*
	Control	1.05 ± 0.63	1 (1–1)		1.20 ± 0.93	1 (1–1)		1.20 ± 0.93	1 (1–1)		*0.95*
Sleep latency	Experimental Control	0.81 ± 0.07	1 (0–1)	*0.16*	0.31 ± .0.47	1 (0–0)	*0.03*	0.39 ± 0.18	1 (0–0)	0.003	*0.002*
		0..54 ± 0.61	1.5 (0–1)		0.71 ± 0.78	1 (0–1)		0.71 ± 0.78	1 (0–1)		*0.11*
Habitual sleep efficiency	Experimental	1.50 ± 1.52	1 (0–3)	*0.41*	0.46 ± 0.62	0 (0–1)	*0.04*	0.15 ± 0.36	0 (0–0)	0.03	*< 0.001*
	Control	1.80 ± 1.49	3 (0–3)		1.31 ± 1.43	3 (0–3)		0.48 ± 0.70	0 (0–1)		*< 0.001*
Sleep disturbance	Experimental	1 ± 0.35	1 (1–1)	*0.57*	0.37 ± 0.49	1 (0–1)	*< 0.0001*	0.12 ± 0.33	1 (0–0)	< 0.0001	*< 0.001*
	Control	1.05 ± 0.48	1 (1–1)		0.97 ± 0.45	1 (1–1)		0.97 ± 0.48	1 (1–1)		*1*
Daytime dysfunction	Experimental	0.84 ± 0.8	1 (0–1)	*0.77*	0.34 ± 0.54	0 (0–0)	*< 0.0001*	0.12 ± 0.33	0 (0–0)	< 0.0001	*< 0.001*
	Control	0.82 ± 0.51	1 (1–1)		1.20 ± 0.79	1 (1–2)		1.14 ± 0.73	1 (1–2)		*0.02*
Global PSQI score	Experimental	6.46 ± 2.43	6.5 (5–7)	*0.83*	5.37 ± 1.97	5.5 (4–7)	*< 0.0001*	4.87 ± 2.21	5 (4–6)	< 0.0001	*< 0.001*
	Control	6.31 ± 2.62	6 (5–8)		7.62 ± 2.51	8 (6–9)		2.70 ± 7.57	8 (6–9)		*0.03*

* Mann-Whitney

** Friedman test

^a^ Mean ± standard deviation, Expected range for each variable: (0–3; 0 = best result; 3 = worst result)

^b^ Median (CI: Confidence Interval): correspond to their respective 95% CI

**Table 3:** Effect of the Aerobic exercise intervention on fatigue as measured by the Multidimensional Fatigue Inventory questionnaire

Variable	Groups	Baseline		4 weeks of Intervention		8 weeks of Intervention		Friedman
		Mean ± SD	p-value*	mean ± SD	p-value*	mean ± SD	*p*-value*	p-value**
General fatigue	Experimental	12.68 ± 0.93	*0.49*	10.12 ± 1.60	*< 0.001*	8.59 ± 1.62	< 0.001	*< 0.001*
	Control	12.54 ± 0.61		12.14 ± 1.19		12.17 ± 0.74		*0.1*
Physical fatigue	Experimental	12.46 ± 0.91	*0.66*	9.84 ± 1.54	*< 0.001*	8.25 ± 1.68	< 0.001	*< 0.001*
	Control	12.37 ± 0.73		15.97 ± 1.11		12.08 ± 0.70		*0.13*
Mental fatigue	Experimental	12.37 ± 0.94	*0.37*	9.68 ± 1.59	*< 0.001*	8.09 ± 1.69	< 0.001	*< 0.001*
	Control	12.17 ± 0.74		11.82 ± 1.20		11.74 ± 0.74		*0.09*
Reduced activity	Experimental	12.25 ± 0.95	*0.17*	9.18 ± 2.32	*< 0.001*	7.90 ± 1.71	< 0.001	*< 0.001*
	Control	11.94 ± 0.63		11.60 ± 1.21		11.54 ± 0.70		*0.04*
Reduced motivation	Experimental	11.96 ± 0.86	*0.49*	9.28 ± 1.54	*< 0.001*	7.46 ± 1.79	< 0.001	*< 0.001*
	Control	11.80 ± 0.58		11.37 ± 1.16		11.54 ± 0.70		*0.06*
Global MFI score	Experimental	61.87 ± 4.38	*0.27*	48.40 ± 7.77	*< 0.001*	40.62 ± 8.27	< 0.001	*< 0.001*
	Control	60.85 ± 2.86		48.40 ± 7.77		58.91 ± 3.35		*0.007*

*Mann-Whitney

** Friedman test

## Discussion

Our results demonstrated that 8 weeks of aerobic exercise was able to improve all sleep components, although improvement in sleep duration was not significant after 4 weeks of intervention. It seems that increasing the intensity of exercise from mild to moderate enhanced all aspects of sleep quality. Also, after four and 8 weeks of intervention, an aerobic exercise decreased the total fatigue score and its components. Since both control and experimental groups underwent midterm exams, the exercise intervention was also able to positively affect the sleep quality during the exams period. The preventive role of exercise in this regard should also be emphasized due to its clinical applications.

The majority of the studies show positive impact of aerobic exercise on sleep quality in students, with one single exception. Studies show that exercise significantly improved sleep quality among students [23, 24]. However in an Iranian quasi-experimental study, at least three sessions per week for 3 months of intervention in 48 students (24 female and 24 male) had no significant influence on sleep quality in either gender [20]. The difference in findings can be attributed to confounding factors (i.e., marital and employment status, household type, intake of herbal medicine, and exercise type which were not disclosed in the study.

The varying intensity levels of physical activity may affect sleep quality differently. Moderate-intensity exercise is generally defined at 65–70% of maximal heart rate [36]. Our results revealed that increasing the intensity of exercise from mild to moderate after 4 weeks could improve sleep quality. According to consultation with sports science expert, the intervention should be designed from mild to moderate activity for participants who did not exercise regularly. Our results revealed that increasing the intensity of exercise from mild to moderate after 4 weeks could improve sleep quality. Consistent with our results, several studies report the benefits of moderate physical activity on sleep [18, 23, 37–40]. A systematic review also showed that moderate exercise has promising outcomes on sleep quality [41]. Thus, examining exercise intensity is imperative to understanding the linkage between physical activity and sleep.

Time of day is also a critical components of exercise [39]. In the present study, early evening exercise increased sleep quality. Morin et al. similarly found that physical activity early in the evening improves the quality of sleep [42]. Results from a systematic review and meta-analysis showed that moderate evening exercise may have positive impact on sleep [43]. Further studies are required to examine the effects of exercise at different times of day on sleep quality.

Concerning the frequency of physical activity, consistent with our results, some studies found that regular physical activity can lead to a more efficient sleep period [44, 45]. A survey reported a positive association between increased frequency of exercise and sleep in young adults [43]. Wu et al. did not observe an association between physical activity and better sleep quality [46]. In a study surveying athletes, 1 day without physical activity had harmed subsequent sleep [47], concluding the benefits of frequent engagement in physical activity on sleep.

According to our findings, four and 8 weeks of aerobic exercises also improved fatigue level and its components in university students. De. Veris’s study revealed significant beneficial effects of aerobic exercises on fatigue level of university students [48]. In one study of multiple sclerosis patients, aerobic exercises significantly reduced fatigue level compared the control group [28]. Physical activity in individuals with pulmonary arterial hypertension could also reduce fatigue severity [14].

Generally, the results of various surveys show that physical activity may decrease fatigue level and promote sleep quality through various mechanisms. Perhaps the most possible mechanism could be attributed to anxiety reduction through exercise [49]. Sleep might also serve as a down-regulation function. During the sleep period, decrease in body temperature occurs as a normal biological process. Given that physical activity increases body temperature, the body seeks to regain its homeostasis and thereby uses the same processes during the sleep period to decrease body temperature by dilation of blood vessels and increased blood flow to the peripheral regions of the body [50]. This process therefore acts as a catalyst of sleep initiation. Moreover, studies showed the anti-depressant effects of exercise which mediated by nightly increases of non-rapid eye movement (NREM) and decreases in REM sleep as well as alteration of Slow-wave sleep (SWS) through temperature elevation [49, 51]. Exercise is often advocated for physiological health benefits and could improve mental health by affecting fatigue level [52]. Additionally, physical activity could impact sleep through the cardiac system by accelerating the re- entrainment to a shifted light–dark cycle [49].

## Conclusions

Poor quality sleep and daily fatigue are prevailing among dormitory students, especially female students. Therefore, non-pharmacological approaches are recommended due to their low-risk and accessibility. Aerobic exercise activities is one of the suggested strategies. Given the findings of the present study, performing a regular aerobic exercise with increasing its intensity is recommended as a practical approach to improve fatigue and sleep quality in non-athletic students.

### Limitations

The inconsistencies in daily sleep hours and daily routines among study participants need to be considered during the interpretation of the results.

## Data Availability

The datasets used and/or analyzed during the current study are available from the corresponding author on reasonable request.

## Abbreviations

AASM: American Academy of Sleep Medicine
PSQI): Pittsburgh Sleep Quality Index
MFI-20: Multidimensional Fatigue Inventory
IUMS: Iran University of Medical Sciences
IRCT: Iranian Registry of Clinical Trials
SWS: Slow-wave sleep
